# Developing Extracellular Matrix Technology to Treat Retinal or Optic Nerve Injury

**DOI:** 10.1523/ENEURO.0077-15.2015

**Published:** 2015-10-08

**Authors:** Tanchen Ren, Yolandi van der Merwe, Michael B. Steketee

**Affiliations:** 1 Department of Ophthalmology, School of Medicine, University of Pittsburgh, Pittsburgh, Pennsylvania 15216; 2 McGowan Institute for Regenerative Medicine, University of Pittsburgh, Pittsburgh, Pennsylvania 15219; 3 Department of Bioengineering, Swanson School of Engineering, University of Pittsburgh, Pittsburgh, Pennsylvania 15260; 4 Center for Neuroscience, University of Pittsburgh, Pittsburgh, Pennsylvania 15216

**Keywords:** axon regeneration, ECM, immunotherapy, regenerative medicine, retinal ganglion cell

## Abstract

Adult mammalian CNS neurons often degenerate after injury, leading to lost neurologic functions. In the visual system, retinal or optic nerve injury often leads to retinal ganglion cell axon degeneration and irreversible vision loss. CNS axon degeneration is increasingly linked to the innate immune response to injury, which leads to tissue-destructive inflammation and scarring. Extracellular matrix (ECM) technology can reduce inflammation, while increasing functional tissue remodeling, over scarring, in various tissues and organs, including the peripheral nervous system. However, applying ECM technology to CNS injuries has been limited and virtually unstudied in the visual system. Here we discuss advances in deriving fetal CNS-specific ECMs, like fetal porcine brain, retina, and optic nerve, and fetal non-CNS-specific ECMs, like fetal urinary bladder, and the potential for using tissue-specific ECMs to treat retinal or optic nerve injuries in two platforms. The first platform is an ECM hydrogel that can be administered as a retrobulbar, periocular, or even intraocular injection. The second platform is an ECM hydrogel and polymer “biohybrid” sheet that can be readily shaped and wrapped around a nerve. Both platforms can be tuned mechanically and biochemically to deliver factors like neurotrophins, immunotherapeutics, or stem cells. Since clinical CNS therapies often use general anti-inflammatory agents, which can reduce tissue-destructive inflammation but also suppress tissue-reparative immune system functions, tissue-specific, ECM-based devices may fill an important need by providing naturally derived, biocompatible, and highly translatable platforms that can modulate the innate immune response to promote a positive functional outcome.

## Significance Statement

Failed regeneration in CNS neurons is increasingly linked to the innate response of the immune system to injury, which leads to tissue destructive inflammation and scarring. Extracellular matrix (ECM) technology has been widely successful clinically in modulating the innate response of the immune system to reduce inflammation and to increase positive tissue remodeling, over scarring. ECM technology is now being developed to treat CNS injuries. Here we discuss recent advances in developing ECM technology in two platforms, an injectable ECM hydrogel and an ECM hydrogel and polymer “biohybrid” sheet. Unlike traditional immunosuppressive treatments that also suppress beneficial immune system functions, ECM-based devices offer a natural biocompatible platform for modulating the innate immune response to promote functional CNS tissue repair.

## Introduction

Approximately 2.5 million cases of ocular trauma are reported annually in the United States, with about 50,000 of these cases resulting in permanent vision loss at an estimated lifetime cost of approximately $900,000 per person according to the National Federation of the Blind (2013). Additionally, ∼80 million people in the United States experience eye-blinding diseases. Worldwide, ∼285 million people are visually impaired, and this number is predicted to increase with increasing longevity (Pascolini and Mariotti, 2012). However, we lack a clinically relevant regenerative medicine approach to promoting constructive tissue remodeling in the neural tissues of the eye, the retina, and the optic nerve. This review discusses developing extracellular matrix (ECM) hydrogel technology to promote constructive tissue remodeling, over scarring, after trauma or disease by modulating the innate immune response to injury. Tissue-specific ECM hydrogels may also provide a more biologically relevant delivery platform for existing retinal and optic nerve repair technologies, like delivering neurotrophic factors, stem or progenitor cells, or antioxidants, among others. Though this review focuses primarily on the retinal ganglion cells (RGCs), microglia, and macrophages, the potential benefits of ECM technology are widely applicable to other neural and glial populations throughout the CNS.

**Figure 1.**
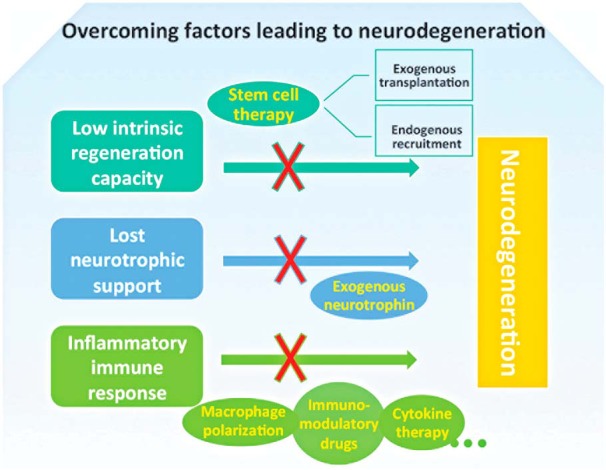
Numerous barriers must be overcome to prevent neural degeneration. The low intrinsic regeneration capacity of RGCs, lost neurotrophic support, and an inflammatory immune response are three major factors leading to neurodegeneration. Therapies overcoming these barriers are showing promise in preclinical and clinical models, including stem cell delivery from both exogenous and endogenous sources; neurotrophin delivery; and immunomodulatory therapies using macrophage polarization, immunomodulatory drugs, and cytokines.

In adult mammalian CNS neurons, failed axon regeneration remains a persistent problem due to the variety of factors prohibiting axon regeneration. In the visual system, injury to RGC axons often leads to progressive RGC axon degeneration, and ultimately to RGC death and permanent vision loss. The inability of CNS neurons to regenerate injured axons is due to multiple intrinsic and injury-induced factors that suppress axon regeneration, including poor intrinsic axon growth ability (Goldberg et al., 2002), altered organelle dynamics (Lathrop and Steketee, 2013), lost neurotrophic support, (Mansour-Robaey et al., 1994), glial expressed inhibitory molecules like Nogo-A, myelin-associated glycoprotein, and oligodendrocyte myelin glycoprotein (McKeon et al., 1999; Tang et al., 2001), and the innate immune response (Horn et al., 2008; Fig. 1), among others.

After injury, RGCs can regenerate axons over long distances, if provided a suitable substrate like a peripheral nerve graft, to reinnervate visual centers in the rodent brain (Vidal-Sanz et al., 1987). In rodent models, transected RGC axons initially display transitory axonal sprouting, indicating that some intrinsic capacity for regeneration exists, and then ∼90% undergo apoptotic cell death within 14 d (Berkelaar et al., 1994). After injury, activated glia, both in the retina and in the optic nerve, produce pro-regenerative neurotrophic factors (Hauk et al., 2008), indicating that the glial population also has some capacity for supporting axon regeneration in the adult mammalian CNS. However, reactive astrocyte and other glial cell-mediated remodeling of the ECM (Fisher et al., 2005; Lewis et al., 2010; Luna et al., 2010; Kayama et al., 2011) ultimately produces a glial scar containing proteoglycans (Silver and Miller, 2004), including neurocan, brevican, phosphacan, and versican (Jones et al., 2003; Butt et al., 2004), and myelin-derived molecules that inhibit axon growth in CNS tissues (Trimmer and Wunderlich, 1990; Schwab, 2010). Thus, in developing new regenerative therapies for CNS injuries, a more comprehensive “tissue-level” approach is required that includes modulating glial cell activation and ECM remodeling to suppress scarring.

Combinatorial approaches, targeting one or more axon growth-inhibiting factors by molecular and/or genetic manipulations, can improve RGC survival and increase axon regeneration in the visual system (Kurimoto et al., 2010). In optic nerve crush studies, full-length axon regeneration was reported in mice treated with combinatorial therapies that increase intrinsic axon regeneration potential, indicating that injured RGC axons can regenerate through the “host” optic nerve to reinnervate the brain. Though the percentage of neurons that regenerated sufficiently to reinnervate the brain was low, encouragingly, some functional recovery was reported (de Lima et al., 2012). Additionally, studies using peripheral nerve grafts and bioengineered neural bridges have shown that transected RGC axons can also regenerate over long distances through nonhost tissues (Berry et al., 2008) and biomaterials (Wittmer et al., 2011). In some cases, transected RGC axons regenerated sufficiently to reinnervate the brain. However, these studies also reported that a low percentage of axons regenerated successfully (Bray et al., 1987). Instead of trying to target numerous growth-inhibiting factors, which may lead to a never-ending series of complications due to off-target effects, or relying on foreign tissue or bioengineered neural bridges, which may not be compatible in the long term and are difficult to envision routing properly to the numerous visual centers in the brain, a more logical approach may be to treat CNS injury at the tissue level by altering the default healing response to injury.

## The innate immune response and CNS regeneration

The default healing response in the CNS is closely linked to the timing and nature of the innate immune response to injury (Koh and DiPietro, 2011; Godwin et al., 2013). After CNS trauma, damaged and dying cells release chemokines and other molecules (Popovich and Longbrake, 2008; Brinkmann and Zychlinsky, 2012) that modulate multiple innate immune system cells, including neutrophils (Schnell et al., 1999; Donnelly and Popovich, 2008), macrophages (Soares et al., 1995), and microglia (Hains and Waxman, 2006), among others. Studies from animals that can regenerate CNS tissues, including retina, optic nerve, and brain, indicate that the temporal and spatial organization of macrophage phenotypes is a critical determinant in the overall healing response (Shechter et al., 2009, 2013). For example, in vertebrates like the axolotl, the temporal and spatial patterning of macrophage phenotypes appears to determine whether injured tissues are repaired functionally or whether scar tissue is formed (Godwin and Rosenthal, 2014).

Macrophages and microglia, the resident macrophage-like cells in the CNS, alter their phenotypes along a spectrum, ranging from the classically activated proinflammatory, M1-like phenotype to the alternatively activated anti-inflammatory, M2-like phenotype (Martinez and Gordon, 2014). This spectrum of phenotypes and their spatial and temporal ratios to one another play distinct roles in the healing process in most vertebrates, determining whether a tissue forms scar tissue or whether a tissue remodels to preserve or restore function. M1-like and M2-like macrophages can generally be distinguished by assessing cytokine, receptor, and enzyme expression levels. Classic M1-like markers include, but are not limited, to proinflammatory markers like interleukin (IL)-1β, IL-6, IL-12, tumor necrosis factor-α, inducible nitric oxide synthase, and C-X-C motif chemokine 10, while alternatively activated M2-like markers include transforming growth factor-β (TGF-β), IL-10, IL-1 receptor antagonist, arginase-1, CD206, and CD163 (Kawamura et al., 2009; Hao et al., 2012; Martinez and Gordon, 2014). Of note, M1- and M2-like markers can vary significantly depending on species, tissue, or organ, and the nature of the injury or pathogen. Thus, careful consideration of multiple markers must be used to identify macrophage phenotypes. Generally, proinflammatory M1-like cells produce high levels of oxidative metabolites (e.g., nitric oxide and superoxide) and proinflammatory cytokines, which are essential for host defense and tumor cell killing (Sica and Mantovani, 2012). However, these factors also cause secondary tissue damage, expand the injury area, and contribute to increased scarring (Mantovani et al., 2004). In contrast, M2-like macrophages are generally thought to be anti-inflammatory and promote functional tissue remodeling (Kigerl et al., 2009). Immunodepleting M1-like macrophages in adult mammals can reduce scarring and improve tissue preservation and functional outcomes. Conversely, cytokines like IL-4 or IL-13 promote an M2-like phenotype (Mokarram et al., 2012), which is thought to reduce tissue-destructive inflammation and to increase functional tissue remodeling (Sica et al., 2006). In CNS tissues, M1 macrophages are neurotoxic and possess only moderate axon growth-promoting effects (Kigerl et al., 2009), whereas M2 macrophages are non-neurotoxic and can promote long-distance axon growth, even in the presence of growth-inhibitory molecules like chondroitin sulfate proteoglycans (CSPG) or myelin (Kigerl et al., 2009). The M2 phenotype has been implicated in tissue repair through cytokine secretion that in turn supports new ECM deposition and tissue remodeling during the wound-remodeling stage (Mosser and Edwards, 2008). Thus, after CNS injury, immunomodulatory therapeutics designed to positively modulate the ratio between M1-like and M2-like macrophages are a logical approach to improving CNS repair.

## Extracellular matrix technology

**Figure 2.**
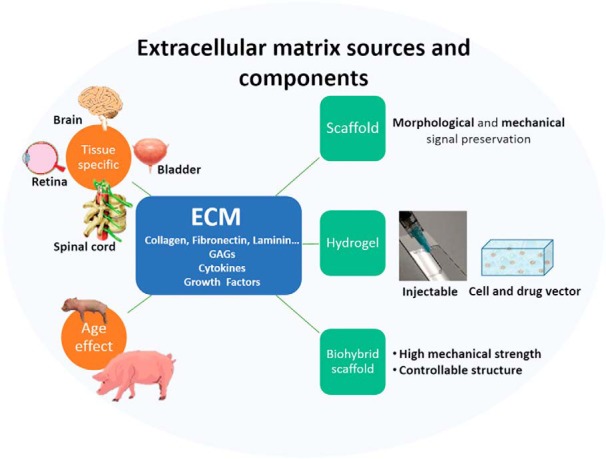
ECMs can be derived from different animal tissues or organs, each with a unique compliment of proteins, carbohydrates, extracellular matrix molecules, growth factors, and cytokines. The ECM is a flexible platform that can be used as a natural material bioscaffold, an injectable hydrogel, or combined with polymeric materials to form biohybrid devices with controllable mechanical and biochemical properties. Each form can be augmented with cells or other bioactive molecules to improve the healing response.

How can we modulate microglia and macrophage phenotypes to promote a more favorable healing outcome in CNS tissues? Extracellular matrix technology is an attractive candidate. Naturally derived ECM bioscaffolds have been shown to modulate the innate immune response in wide-ranging applications throughout the body. ECM technology uses ECM bioscaffolds derived by decellularizing various healthy mammalian tissues or organs primarily from porcine or equine sources (Gilbert et al., 2006; Badylak, 2007; Fig. 2). ECM bioscaffolds maintain many of the bioactive molecules specific to the native tissue, including collagens, glycosaminoglycans, laminins, and growth factors (Crapo et al., 2012; Turner and Badylak, 2013), that are unavailable in synthetic materials. When properly prepared, ECM does not produce an adverse immune response (Keane et al., 2012) and is highly translatable clinically. Over 4 million patients have been treated with >60 Food and Drug Administration-approved, ECM-based products used to treat injuries in varied tissues, including skin, heart, esophagus, bladder, muscle, bone, and peripheral nerves, among others (Badylak et al., 2005; Badylak, 2007; Valentin et al., 2010; Wolf et al., 2012). However, few initial studies have analyzed whether ECM technology can modulate the default healing response in the brain or spinal cord (Liu et al., 2011; Bible et al., 2012; Zhang et al., 2013), and applying ECM technology to retinal or to optic nerve injuries has been virtually unexplored.

ECM bioscaffolds can modulate several components of the default healing response relevant to constructive CNS tissue remodeling. For example, porcine small intestine ECM (SIS-ECM) bioscaffolds induce angiogenesis in an esophageal resection and repair model in dog (Badylak et al., 2000), and both angiogenesis and neurogenesis in a murine model of volumetric skeletal muscle loss (Sicari et al., 2012b). SIS-ECM bioscaffolds can also promote innervation in a rodent abdominal wall reconstruction model (Agrawal et al., 2009). ECM degradation products derived from digesting urinary bladder ECM can direct both progenitor cells and resident tissue-derived cells to repopulate the injury site in a mouse model of digit amputation (Agrawal et al., 2011, 2012). Decellularized peripheral nerve ECM can stimulate axon regeneration in the rodent sciatic nerve (Vasudevan et al., 2014) as well as in human clinical studies in part by stimulating Schwann cell migration and myelination (Karabekmez et al., 2009; Cho et al., 2012). ECM-based hydrogels can reduce glial activation in rodent CNS trauma models (Lin et al., 2009; Khaing et al., 2011) with reports of improved neurologic function (Huang et al., 2012). Finally, ECM bioscaffolds also possess antimicrobial activities (Brennan et al., 2006; Medberry et al., 2012), which are critical in any successful wound-healing scenario.

However, modulating the innate response of the immune system to injury is increasingly recognized as the critical factor necessary to bias the default healing response toward site-appropriate, functional tissue remodeling. ECM-derived factors are hypothesized to modulate the innate immune response by regulating the spatial and temporal ratios between M1-like and M2-like macrophage and microglia phenotypes. Naturally derived ECM bioscaffolds have been shown to increase M2-like, pro-regenerative macrophages at the ECM implantation site in muscle defect models based on both immunohistochemical and quantitative PCR for M1-like and M2-like macrophage markers (Badylak et al., 2008; Brown et al., 2009, 2012). However, whether ECM can similarly regulate M1/M2 phenotypes in infiltrating macrophage and resident microglia, which differ considerably from monocyte-derived macrophages (Cherry et al., 2014), in the CNS is largely unknown. Depleting or inhibiting M1 macrophage activity has long been recognized as being neuroprotective in the CNS (Giulian and Robertson, 1990; Gris et al., 2004). In a rodent traumatic brain injury (TBI) model, urinary bladder matrix (UBM) injected into the brain did not detectably activate microglia or astrocytes, while decreasing the lesion volume and increasing functional recovery (Zhang et al., 2013). In a rodent spinal cord injury model, M2-like macrophage-derived factors were shown to reduce astrocyte activation, which in turn reduced M1-like macrophage infiltration via a putative feedback mechanism (Haan et al., 2015). Since the glial scar contains inhibitory molecules like CSPGs, which not only prohibit axon regeneration (McKeon et al., 1999) but also appear to polarize macrophages toward an M1 phenotype (Bartus et al., 2014; Didangelos et al., 2014), the glial scar appears to promote proinflammatory signaling indefinitely at the lesion site (Kigerl et al., 2009). Thus, the antigliotic and immunomodulatory properties of naturally derived ECM bioscaffolds should be further explored in the CNS as a preemptive strategy for reducing scarring.

How do ECM bioscaffolds modulate the default healing response at a signaling level? ECM-induced changes in the default healing response are attributed to both chemotactic and chemotrophic factors, released during ECM degradation (Reing et al., 2009), that direct site-appropriate cellular migration and differentiation. In turn, appropriately differentiated cells feedback positively by exerting site-appropriate changes in the extracellular matrix in a process termed “dynamic reciprocity” (Bissell and Barcellos-Hoff, 1987; Berthiaume et al., 1996; Nelson and Bissell, 2006). After ECM is applied *in vivo*, ECM bioscaffolds are rapidly invaded and degraded by macrophages and other immune cells. During degradation, factors are released, including growth factors that contribute to healing, like VEGF (Sage, 1997; Hodde et al., 2001), TGF-β, PDGF, BMP4, and bFGF (McDevitt et al., 2003; Badylak, 2004). Recent studies have demonstrated that biologically active ECM degradation products are released from collagen, laminin, and fibronectin molecules (Davis et al., 2000; Adair-Kirk and Senior, 2008; Brennan et al., 2008; Crisan et al., 2008; Reing et al., 2009) and from angiogenic proteins (Sage, 1997; Hodde et al., 2001). These so-called “matricryptic peptides” can recruit endogenous stem cells and direct their migration (Agrawal et al., 2011), proliferation (Reing et al., 2009), and differentiation into site-appropriate cell types. For example, peptides generated by enzymatic degradation of urinary bladder ECM identified a C-terminal telopeptide of collagen IIIα that can direct stem cell chemotaxis *in vitro* and attract Sox2^+^/Sca1^+^/Lin^−^ progenitor cells *in vivo* in a mouse digit amputation model (Agrawal et al., 2011). Moreover, in a dog musculoskeletal model, ECM-derived scaffolds have been shown to recruit CD133^+^ myogenic progenitor cells (Turner et al., 2012) as well as Sca1^+^/PW1^+^ interstitial muscle stem cells (Perniconi et al., 2011). Whether similar mechanisms can direct CNS stem cell populations remains to be determined but is an ongoing area of investigation.

**Figure 3.**
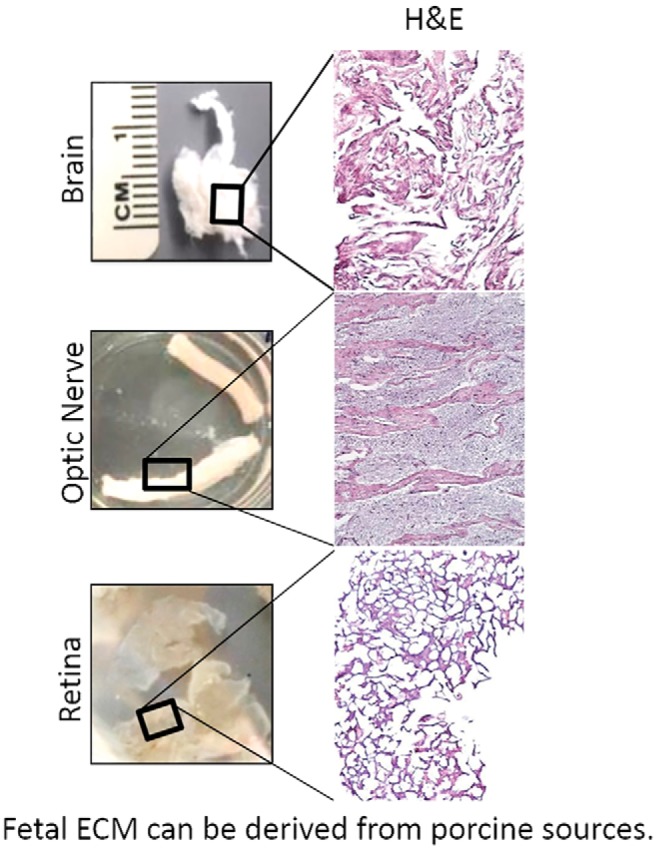
Images and H&E staining of different decellularized CNS ECMs derived from fetal porcine tissues.

**Figure 4.**
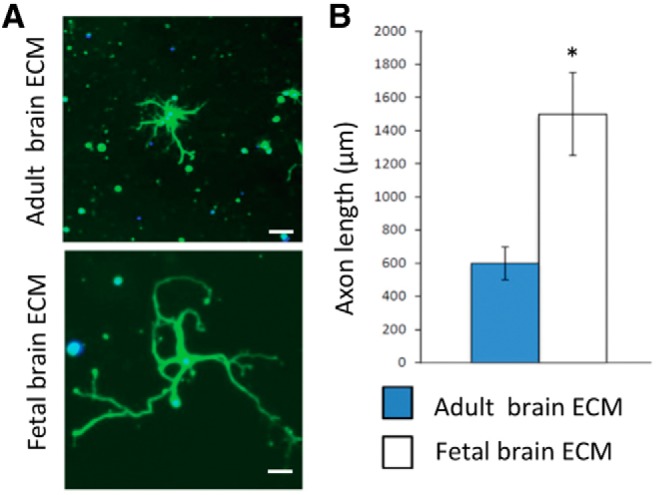
RGCs grow longer processes in ECM derived from younger, homologous tissue sources. ***A***, Fluorescence images of purified primary rat RGCs cultured in adult or fetal brain ECMs. ***B***, Quantification: *p <* 0.001, ANOVA.

Both age and tissue type are important factors with ECM-derived from younger, tissue-matched sources, often achieving increased progenitor cell recruitment and enhanced ECM-mediated alteration of the default immune response toward a pro-repair M2 macrophage phenotype (Brennan et al., 2008; Sicari et al., 2012a). Thus, tissue-matched ECM bioscaffolds, like porcine brain, optic nerve, or retinal ECMs, may be good candidates for modulating the innate immune response in CNS-specific, brain, spinal cord, retinal, or optic nerve injuries. Furthermore, recent advances in decellularization techniques have permitted ECM to be derived from delicate tissues previously not decellularizable using established protocols, including fetal brain, optic nerve, and retina (Fig. 3). The ability to use hydrogels derived from fetal, tissue-matched sources, particularly in delicate CNS tissues like the retina, is an exciting and open area for investigation. Preliminary *in vitro* studies indicate fetal, tissue-specific ECMs can increase retinal ganglion cell survival and axon regeneration significantly over adult tissue*-*derived versions (Fig. 4).

## Modulating inflammation is critical to a positive outcome in CNS tissues like retina and optic nerve

Inflammation plays a central role in the healing outcome and in the rate of disease progression. Inflammation exerts both positive and negative effects on RGC regeneration. After incisional or penetrating trauma to the retina, cells die in all retinal layers adjacent to the wound (Sipperley et al., 1978; Turner et al., 1986). Similarly, all photoreceptors adjacent to the wound die by apoptosis or programmed necrosis. Retinal cell death is accompanied by the rapid onset of glial activation (Yoshida et al., 1995; Fisher et al., 2005). Within 30 min of injury, cell cycle-related transcription factors that regulate astrocyte proliferation, like c-Fos and Jun-B, are upregulated (Fisher et al., 2005). Within 3 d, glial fibrillary acidic protein and proliferating cell nuclear antigen, markers of activated and proliferating glial cells, are upregulated (Yoshida et al., 1995; Vazquez-Chona et al., 2004). Activated glia in the retina and in the optic nerve produce multiple pro-regenerative neurotrophic factors (Hauk et al., 2008; Ahmed et al., 2010). However, reactive astrocytes also contribute to injury-induced ECM remodeling; altered synaptic connectivity in the retinal layers; and altered photoreceptor cellular organization, function, and synaptic connectivity (Fisher et al., 2005; Lewis et al., 2010; Luna et al., 2010; Kayama et al., 2011). Various proinflammatory stimuli, including lens injury, intravitreal injection of zymosan (macrophage activator) or stem cells, and intravitreal peripheral nerve grafting, can all reduce injury-induced RGC death (Berry et al., 1996; Leon et al., 2000; Yin et al., 2003; Mead et al., 2013), presumably by modulating the nature of the inflammatory response. For example, after lens injury and intravitreal zymosan injection, retinal astrocytes and Müller cells release CNTF, which supports axon regeneration (Muller et al., 2007). Immunomodulation combined with endogenous or exogenous growth factors can enhance RGC regeneration synergistically (Kerschensteiner et al., 2003; Lorber et al., 2008). After incision-induced trauma to the retina, some types of inflammation appear to reduce retinal cell death and increase RGC axon regeneration (Berry et al., 2008). Immunosuppressive agents like corticosteroids have also been used to treat intermediate and posterior segment uveitis with some success. Corticosteroids can reduce inflammation and alleviate other structural complications. However, complete suppression of the immune system leads to secondary complications (Thiyagarajan et al., 2013) that may actually prohibit long-term recovery in the CNS. In general, inflammation due to retinal trauma promotes an M1-like phenotype, which decreases RGC survival and increases scarring (Cruz-Guilloty et al., 2013; He and Marneros, 2013), emphasizing the importance of identifying the type and the scope of the inflammatory response, as well as highlighting the need for readily available and versatile immunomodulatory therapeutic platforms.

Can ECM-based immunomodulatory devices fill this need in the CNS, for example by slowing or halting retinal disease progression? Increasingly, microglial activation is implicated in retinal disease pathogenesis, including glaucoma (Yuan and Neufeld, 2001), diabetic retinopathy (Zeng et al., 2008), age-related macular degeneration (AMD; Gupta et al., 2003), and retinitis pigmentosa (RP; Gupta et al., 2003), among others. Transplantation of human induced pluripotent stem cell-derived retinal cell types has been proposed as a potential treatment for AMD and RP. However, this treatment strategy needs to be optimized with regard to characterizing and preparing donor cells (Ramsden et al., 2013). Current clinical immunotherapies can inhibit microglial activity (Yrjänheikki et al., 1998). However, such strategies can also disrupt microglial regulation of CNS homeostasis (Edan et al., 2013). Thus, a more biocompatible immunotherapy like ECM may be advantageous under certain conditions alone or as an adjunct therapy. For example, in AMD, macrophages penetrate the interphotoreceptor matrix and polarize toward an M1-like phenotype, which is recognized as a key factor in dry AMD pathogenesis (Cruz-Guilloty et al., 2013). In retinal diseases, can ECM hydrogels be used to modulate microglial activation directly or in combination by delivering other therapeutics in a natural, injectable, biodegradable device? And, if so, can sustained modulation be achieved in various diseases?

## ECM technology can mitigate secondary trauma

Primary mechanical injuries are often followed by additional tissue destruction and expanded scarring due to the inflammatory response. In the visual system, secondary ocular trauma in the retina or in the optic nerve can rapidly increase the injury area and lead to increased vision loss. One of the most detrimental injury conditions is during warfare, where significant delays in ocular treatment are common. In modern warfare, blast injuries are the most common wounded-in-action injuries accounting for ∼60% of all injuries as of July 2009, with up to 40% of blast injuries expressing concomitant eye injuries. To treat ocular trauma due to penetrating injuries and intraocular foreign bodies, the current standard of care requires a vitreoretinal service and a microsurgical operating suite, which is generally not possible on the battlefield or in Level III combat support hospitals. In a combat support hospital, patients do receive an eye evaluation and primary surgical repair by an experienced ophthalmologist (Weichel and Colyer, 2008), often within hours of injury. However, in cases where a microsurgical suite and a vitreoretinal service are required, patients typically experience a minimum 72-96 h delay before evacuation. These delays are during a critical period in the healing process since secondary inflammatory trauma can increase progressively and is often more damaging than the primary trauma. For example, secondary inflammatory responses to penetrating injuries can contribute to endophthalmitis, which can lead to vision and even eye loss (Lemley and Han, 2007), or proliferative vitreoretinopathy (PVR), which can result in progressive retinal traction, tears, and retinal detachment (Charteris, 1995). The molecular cascades leading to PVR are detectable within hours in rodent models of PVR (Wen et al., 1995; Ozaki et al., 2000; Penn et al., 2006), again emphasizing the need for therapies to suppress secondary inflammation, like ECM-based devices, which are safe, derived from renewable resources, and relatively inexpensive, and can be tailored to the nature and scope of the injury.

## ECM hydrogels are a highly tunable biologic platform

To address this problem, ECM hydrogel technology is being developed in two platforms designed to rapidly mitigate secondary trauma in varied injuries. The first platform is an injectable hydrogel (Medberry et al., 2013) that can be injected into or around the eye, whereas the second platform is a convenient ECM and polymer biohybrid sheet (Hong et al., 2011). These technologies are designed to stabilize retinal or optic nerve injury, reduce scarring, and increase the potential for vision preservation. Both platforms are tunable, both mechanically and biochemically, to deliver drugs, cells, or other therapeutics in a biocompatible, immunomodulatory platform.

## ECM hydrogels

ECM-based hydrogel scaffolds are advantageous because of their potential for minimally invasive delivery. Applying ECM technology to injured neural tissues has been limited, as previously reviewed (Meng et al., 2014), particularly in the CNS where intrinsic regenerative ability is low (Goldberg et al., 2002). However, a number of studies in spinal cord have shown that acellular ECM matrices can integrate within host spinal cords after traumatic brain injury and even improve motor function (Li et al., 2012; Zhang et al., 2012; Liu et al., 2013). UBM has been shown to promote constructive cellular responses to and neuroprotection of injured brain tissues (Zhang et al., 2013). After injection into healthy brains, UBM did not increase microglia accumulation or astrocyte activation, or promote neuronal degeneration. After TBI in rats, UBM treatment reduced lesion volume and myelin disruption, and improved vestibulomotor function. However, cognitive recovery was undetected over the time points analyzed. Recently, Wang et al. (2013) reported similar results following TBI, showing that UBM decreased sensorimotor skill loss, indicating that ECM hydrogels may be a viable treatment option in CNS tissues. In cases of penetrating ocular trauma, local ECM hydrogel injection provides a biocompatible, tunable gel for filling acellular injury-induced defects, recruiting and directing endogenous stem cell localization and differentiation, and modulating the immune system to promote positive tissue remodeling.

## ECM biohybrid bioscaffolds

**Figure 5.**
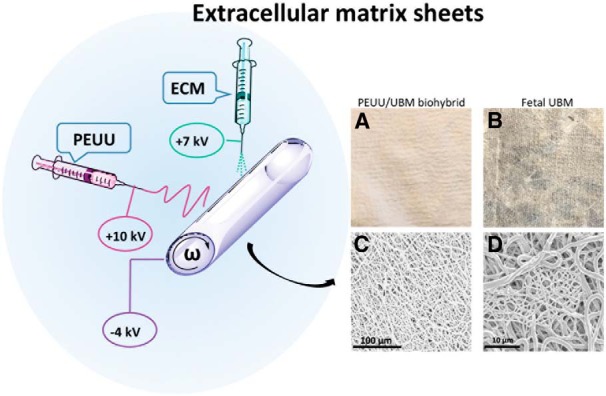
Extracellular matrix in two sheet forms. ECM biohybrid sheets can be made by dual-stream electrospinning. PEUU and ECM electrospinning setup consists of two syringe pumps and two high-voltage power supply units (not shown). A high positive voltage (+7-10 kV) is used to charge the steel capillary containing the polymer or ECM solution, and a high negative voltage (−4 kV) is used to charge the stainless steel mandrel (ω). The mandrel is rotated at 200 rpm with a slow lateral translation over a distance of 15 cm, ***A***, Yielding a tubular, uniform PEUU/ECM sheet. ***B***, Fetal urinary bladder sheet after vacuum pressing. ***C***, ***D***, Scanning electron microscopy showing the random fibers in the PEUU/ECM biohybrid wrap.

Biohybrid scaffolds confer several advantages to ECM hydrogels by combining the mechanical and biochemical tunability of an electrospun polymer sheet with the biologic properties of ECM (Hong et al., 2011; Fig. 5). In regenerative medicine approaches that require bioscaffolds with a higher tensile strength, hydrogels are disadvantageous due to rapid degradation rates and poor mechanical properties. The mechanical strength of ECM hydrogels can be improved by chemical crosslinking (Baiguera et al., 2014). However, crosslinking changes ECM composition, leading to increased risk of inflammation (Valentin et al., 2009), altered degradation rates and products, and altered biological efficacy and effects. Combining ECM hydrogels with a synthetic biocompatible and biodegradable polymer has been shown to be a viable option (Stankus et al., 2008; Yoon and Kim, 2010). Hong et al. (2011) used dual-stream electrospinning to blend synthetic poly(etherurethane urea) (PEUU) polymer and urinary bladder matrix. The resulting ECM biohybrid scaffolds have tunable degradation properties and tunable mechanical properties. For example, electrospun fibers can be aligned to direct axon growth. Kador et al. (2014) created a scaffold with aligned fibers by electrospinning, which guided retinal ganglion axon growth directionally. By integrating ECM hydrogels, biohybrid scaffolds possess higher biocompatibility compared with purely synthetic scaffold materials. In non-CNS models, biohybrid scaffolds have been shown to promote constructive tissue remodeling in ligament, heart, and body wall (Hong et al., 2011; Thayer et al., 2014; Jahnavi et al., 2015). In nervous system tissue repair scenarios, the ability to readily form biohybrid scaffolds into a suturable nerve wrap or a patch to protect exposed tissues makes ECM biohybrid scaffolds attractive devices for both mechanical and biochemical support.

## ECM hydrogels for cellular transplantation

ECM hydrogels provide a tissue-specific, naturally derived platform for delivering transplanted cells in an injectable platform. Transplanting purified retinal progenitor cells or neural stem cells (NSCs) has emerged as a promising therapy for preserving visual function. Intravitreal or optic nerve injections of RGCs, RGC progenitors, or stem cells are minimally invasive and have been shown in some neurodegenerative disease models to be neuroprotective (Klassen et al., 2008; Eveleth, 2013). Transplanted RGCs or stem cells are hypothesized to slow retinal degeneration by modulating multiple prosurvival pathways simultaneously via locally secreted neurotrophic factors and/or via modulation of the intraocular microenvironment (Miyata et al., 2005; Hertz et al., 2014). And clinical trials have been performed to test these hypotheses (Ning et al., 2011). However, RGCs injected into the vitreous integrate randomly without the correct spatial and cellular organization. Moreover, although transplanted RGCs can extend long axons, their orientation is generally not directed toward the optic nerve head, a prerequisite for restoring RGC connectivity to the brain. Transplanting sheets of fetus-derived retinal progenitor cells subretinally can restore some vision in both animal models and humans. In Phase II clinical trials, visual acuity was improved in patients with retinitis pigmentosa or macular degeneration (Seiler and Aramant, 2012), demonstrating that cellular transplantation strategies can preserve retinal function in cases where the ganglion cell connectivity to the brain is intact. Moreover, retinal sheets hold promise for replacing RGCs with the proper cellular organization in the retina. However, similar to single-cell RGC transplantation, retinal sheets cannot replace lost RGCs with the correct retinal and optic nerve organization, and thus cannot currently restore vision loss due to lost RGCs. By using CNS tissue-specific ECM hydrogels, stem or progenitor cells can be encapsulated and then injected precisely within a biocompatible matrix, which may offer greater potential for integration and preservation of function.

In other cases, ECM biohybrid sheets may provide a better option for delivering cells. For example, mesenchymal stem cells (MSCs) can modulate immune and inflammatory effects after injury in both the CNS and peripheral nervous systems (PNS). MSC delivery into the injury site can provide anti-inflammatory, immunomodulatory, and neuroprotective benefits. For example, in dogs with acute spinal cord injury, the injection of MSCs significantly improved functional recovery (Penha et al., 2014). However, further studies have shown these injections cause additional injury due to needle penetration, spinal cord motion during injection, creation of intraparenchymal pressure gradients, and hydrodynamic dissection, instilling deformed cell masses and possible cord ischemia (Donnelly et al., 2012). Other studies, using scaffolds, have shown that fibrin sheets incorporating MSCs demonstrate longitudinal alignment of MSCs and infiltration of host neurites, which are correlated with further improvement of functional outcomes (Hyatt et al., 2014). Several mechanisms have been proposed for the improved axonal regeneration seen after injecting MSCs, including paracrine effects due to the release of trophic factors, including BDNF, NGF, VEGF, FGF-2, TGF-β, and interferon gamma-1 (Mead et al., 2014). By incorporating mesenchymal stem cells into ECM biohybrid sheets, the neuroprotective potential of stem cells is combined with the immunomodulatory benefits of ECM. Moreover, incorporating stem cells into a polymer with tunable biodegradability provides more control over restricting the cells to a defined location, like subretinal implantation or as an optic nerve wrap, while permitting the soluble factors released from both stem cell and macrophage degradation of the ECM components, which have been shown in some systems to have positive synergistic effects with regard to tissue repair (Liu et al., 2011)

Can tissue-specific ECMs provide a more efficacious platform for stem or progenitor cell differentiation and integration? Numerous studies have shown that ECM bioscaffolds can direct site-appropriate cellular differentiation from both endogenous (Horne et al., 2010) and mesenchymal stem cell (Wang et al., 2010) sources. There are numerous examples of tissue-specific ECMs directing tissue-specific cellular phenotype differentiation and function. For example, cartilage-derived ECM can promote chondrogenic differentiation of adipose-derived stromal stem cells (Wang et al., 2014), while cardiac ECM can direct cardiac myocyte differentiation (Xu et al., 2014). Acellular spinal cord scaffolds can promote positive tissue remodeling in spinal cord injury models, similar to MSCs (Liu et al., 2013; Xue et al., 2013). Methods for decellularizing spinal cord (Guo et al., 2010), brain (DeQuach et al., 2011), and optic nerve (Crapo et al., 2012) have been developed from adult and even from fetal sources (Fig. 3). Acelluar CNS ECM scaffolds retain neurosupportive proteins, growth factors in a three-dimensional scaffold. Compared with UBM, CNS-derived ECMs induced PC12 cell migration, while UBM inhibited migration (Crapo et al., 2012). Brain-derived ECM improved neurite growth of neural stem cells (Medberry et al., 2013) and neuronal differentiation of induced pluripotent stem cells. (Crapo et al., 2012). These studies suggest that ECM may provide tissue-specific advantages in CNS regenerative medicine applications and that ECM scaffolds in general may aid functional recovery after retinal disease.

## Non-tissue-specific ECMs can also promote positive CNS repair

Acellular muscle ECM has been used to treat spinal cord hemi-sections in rats. With the help of its parallel tubule structure, sprouting axons grew the full length of the scaffold in a strikingly parallel and linear fashion (Zhang et al., 2012). Loaded with amniotic epithelial cells, acellular muscle scaffolds further promoted nerve fiber sprouting and remyelination, resulting in more functional recovery (Xue et al., 2013). Acellular vessels are also a candidate for nerve regeneration scaffolds. Acellular vessels were used as nerve conduits connecting the two stumps of injured peripheral nerves (Sun et al., 2011). Acellular PNS scaffolds may also be a good choice for optic nerve repair. Sciatic nerves were decellularized with different methods (Gao et al., 2014), and their function in nerve regeneration was evaluated (Li et al., 2012; Gao et al., 2014). Schwann cells can support RGC axon growth, possibly due to neurotrophic factor secretion and/or the neurotrophic cell receptors on the membrane of Schwann cells (Baehr and Bunge, 1989; Hu et al., 2005). Treating ECM with neurotrophic factors can also improve their ability in CNS regeneration (Li et al., 2012).

Cellular viability and retinal integration are improved by using various hydrogel materials, including hyaluronic acid (Gao et al., 2012, Carey et al., 2014), Matrigel (Sharp and Archer, 2015), alginate (Yang et al., 2015), collagen, (Cruz-Guilloty et al., 2013), and fibrin (Meng et al., 2014). Brain ECM hydrogels promoted neuronal differentiation of induced pluripotent stem cells better than Matrigel-coated surfaces (DeQuach et al., 2011). DeQuach et al. (2011) produced brain ECMs and found that neurons derived from human induced pluripotent stem cells plated on the brain matrix express neuronal markers and assume neuronal morphology. Crapo et al. (2014) showed that CNS-derived ECMs show higher ability in inducing neuron differentiation of neuronal stem cells compared with UBM. Liu et al. (2013) developed MSCs that loaded acelluar spinal cord ECM, which promoted long-distance axon regeneration *in vivo*. Moreover, UBM hydrogel has been used as a carrier for NSCs and injected into rodent brain. The transplants reduced neuron/tissue loss and white matter injury, and also significantly ameliorated motor, memory, and cognitive impairments (Wang et al., 2013). Stem cells delivered by UBM hydrogels in a murine stroke model distributed uniformly throughout the lesion cavity instead of integrating into the host parenchyma. Better distribution was associated with better primitive tissue formation (Bible et al., 2012).

## Conclusion

**Figure 6.**
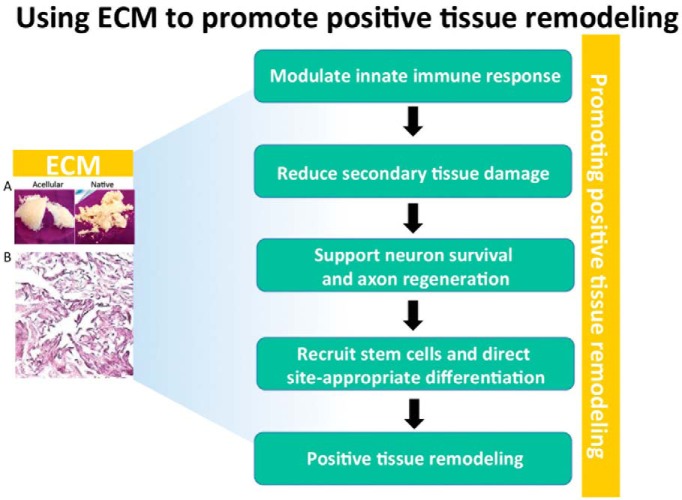
ECM technology can promote positive tissue remodeling by modulating several factors that contribute to the default healing response in the CNS.

ECM-derived hydrogels are natural, biocompatible devices that can modulate inflammation, attract and direct stem cell proliferation and differentiation, and serve as a tunable platform for delivering an almost unlimited combination of genetic, molecular, and cellular therapeutic factors. ECM bioscaffolds have been shown to reduce inflammation and scarring while improving positive tissue reconstruction in tissues throughout the body. We hypothesize that tissue-specific hydrogels can do the same in CNS tissues generally as well as specifically within the retina and the optic nerve (Fig. 6). Ultimately, a highly defined and targeted approach is desired to treat CNS injuries with specificity. Thus, future studies should include further characterization of the bioactive components in the ECM, how various ECM components impact the innate immune response, and, in turn, how soluble and nonsoluble factors act on other cells within the CNS, including the glia, primary neurons, and progenitor and stem cells.

## Synthesis

The decision was a result of the Reviewing Editor Maiken Nedergaard and the peer reviewers coming together and discussing their recommendations until a consensus was reached. A fact-based synthesis statement explaining their decision and outlining what is needed to prepare a revision is listed below.

Synthesis of Reviews: this is potential a nice study, but additional experiments and discussion of the literature is needed.

Reviewer #1

The goal of this study was to develop techniques for precise measurement of blood flow in retinal vessels. The approach involved the study of anesthetized paralyzed rats and visualizing the passage of fRBCs in the retina. Along with baseline data, changes in blood flow to two stimuli (hypercapnia and hyperoxia) were tested. From the data, the authors conclude that the method they describe provides precise and accurate measures of absolute blood flow and velocity in retinal vessels of all sizes. While some elements of the study appear to be new, there are several issues with this study and its presentation.

1. The paper repeatedly states that absolute blood flow was measured. In reality, the description indicates that blood flow was calculated based on other variables that were directly measured. Please be more precise throughout.

2. What are the references listed in the text as: Anonymous, 2012, 2014?

3. Recordings of blood pressure were included in the study, a positive. Along these lines, how stable was blood pressure during the actual measurements of diameter and velocity? Was it affected by interventions like hypercapnia?

4. The main conclusion regarding resting blood flow was based on only an n of 3, and the values are quite variable. For reasons that are not well justified, the authors state this was expected. Variability could be due to a number of factors, and a larger n is typically needed for a new method to be rigourously evaluated and convincing. The authors have not performed a comparison between the current new approach and any other established (gold standard) approach in the same animals. Related to this point, the paper does not provide any kind of serious discussion of how resting values obtained in this study compare with the literature. There are many previous studies that have quantified retinal blood flow (using microspheres and other approaches) in multiple species (eg, rat, cat, rabbit, monkey, pig, etc). For whole tissue and when expressed on a per weight basis, how do flow values in the current and previous studies compare?

5. The authors conclude that the method used here to obtain blood flow is precise (has high precision). This statement is also not entirely convincing. For example, the changes in blood flow are not close to what one would predict based on the diameter changes that are shown. With hypercapnia for example, the change in blood flow is much much less than would be expected based on the diameter change shown (Fig 5). The authors state this was expected because of perfusion pressure changes that likely occurred, but they provide no evidence for such changes or effects in this model. An alternative (that has not been excluded to this point) is that the method for obtaining values for blood flow in the current study is not as precise as is being concluded.

6. How do you interpret the changes in venous diameter? Are these changes active or passive?

7. The manuscript stated that PCO2 was 35-45 mmHg, but only end-tidal CO2 measurements were mentioned. It also says sO2 was greater than 92 mmHg, but states elsewhere that O2 saturation was measured. For both CO2 and sO2, values are presented in mmHg, but it is unclear how that value was derived based on what was measured? During hypercapnia, what level of PCO2 was produced?

Reviewer #2

The presented work documents the use of fluorescent red blood cell monitoring by video microscopy as an approach to assessing retinal blood flow. The approach would appear to provide practically useful information that could be relevant in the context of experimental studies. A few questions arise.

1) It would seem important to emphasize that the methodology provides information only about red cell transit. Issues relating to hematocrit variations within microvasculature have received considerable attention (e.g., Gould and Linninger, Microcirculation 22:1-18, 2015), and any limitations this may place on the interpretation of the results should be considered.

2) There has also been recently published work using video imaging of unlabeled red cells (Ortiz et al., Physiol. Meas. 35:351-367, 2014), so it would seem relevant to compare the limitations and advantages of these approaches.

3) As a very minor point, it might be better to state anesthetic dose in terms of the alpha-chloralose content of the administered complex (10% of total) to permit more direct comparison with past studies.

**Author Response**

Dear Dr. Nedergaard,

As suggested in your letter of 4 February, we have revised our manuscript, “Measurement of retinal blood flow using fluorescently labeled red blood cells.” In all instances, we have followed the advice of the reviewers in revising the manuscript. We thank the reviewers for their helpful comments, which have greatly strengthened the paper. We have made the following changes to the manuscript.

Reviewer #1

The reviewer asks that we be more precise when referring to the method we used in calculating blood flow. To address this, we have made a number of changes to the text to indicate that blood flow was not measured directly but was calculated from multiple measurements.

References that refer to our previous work were listed as Anonymous. This was required by the eNeuro submission guidelines as a way to preserve the double-blind nature of the review process. References to our previous work remain listed as Anonymous in the revised manuscript.

The reviewer asks about the stability of blood pressure in our experimental animals. Blood pressure remained within the normal range (95 - 130 mmHg) during experimentation and did not vary greatly over time. This has been clarified in the manuscript (lines 95-97). Blood pressure was not tracked in real time during hyperoxia and hypercapnia but it remained within the normal range. This is noted in the revised manuscript (lines 141-142).

We thank the reviewer for pointing out that our measurements of resting blood flow are not adequately discussed in the context of the existing literature. We have revised the manuscript to include a more thorough discussion of this issue, comparing our flow values with those from previously published studies (lines 339-347). Our measurement of total retinal blood flow serves as proof of principle, not as a comprehensive analysis of blood flow in the rat. Thus, a large sample size is not necessary. This is clarified in the text (lines 338-341). In addition, as suggested by the reviewer, we have added a paragraph comparing our measurements of blood flow, expressed per unit weight of tissue, to previous studies (lines 346-354).

The reviewer points out that our blood flow measurements may not be precise, particularly as demonstrated in the hypercapnia experiment showing less flow change than expected for a given change in vessel diameter. We have revised the manuscript to provide a more complete explanation of this observation (lines 361-369). In addition, we have removed in all instances the word “precise” from the manuscript.

All evaluations of venous blood flow were done under baseline conditions. The hyperoxic and hypercapnic challenge data came from arterioles only. The manuscript has been revised to clarify this (lines 143, 267, 774).

We thank the reviewer for pointing out errors in documenting blood gas parameters. Indeed, we measured end tidal CO_2_ and not pCO_2_, and sO_2_ and not pO_2_. The manuscript has been revised to correct these errors (line 97). The pCO_2_ value recorded during hypercapnia has also been added (line 141).

Reviewer #2

The reviewer rightly suggests that we should more thoroughly discuss the impact of local hematocrit on our results. The manuscript has been revised to include a discussion of this issue (lines 407-413).

We thank the reviewer for calling to our attention the paper by Ortiz et al. In the revised manuscript, we compare this and other image-processing methods to our own (lines 385-396).

We agree with the reviewer that chloralose dosing can be confusing and have clarified the dosing in the manuscript to better allow for comparison between studies (lines 84-86).

## References

[B1] Adair-Kirk TL, Senior RM (2008) Fragments of extracellular matrix as mediators of inflammation. Int J Biochem Cell Biol 40:1101-1110. 10.1016/j.biocel.2007.12.005 18243041 PMC2478752

[B2] Agrawal V, Brown BN, Beattie AJ, Gilbert TW, Badylak SF (2009) Evidence of innervation following extracellular matrix scaffold-mediated remodelling of muscular tissues. J Tissue Eng Regen Med 3:590-600. 10.1002/term.200 19701935 PMC2787980

[B3] Agrawal V, Siu BF, Chao H, Hirschi KK, Raborn E, Johnson SA, Tottey S, Hurley KB, Medberry CJ, Badylak SF (2012) Partial characterization of the Sox2+ cell population in an adult murine model of digit amputation. Tissue Eng Part A 18:1454-1463. 10.1089/ten.TEA.2011.0550 22530556 PMC3397117

[B4] Agrawal V, Tottey S, Johnson SA, Freund JM, Siu BF, Badylak SF (2011) Recruitment of progenitor cells by an extracellular matrix cryptic peptide in a mouse model of digit amputation. Tissue Eng Part A 17:2435-2443. 10.1089/ten.tea.2011.0036 21563860 PMC3179613

[B5] Ahmed Z, Aslam M, Lorber B, Suggate EL, Berry M, Logan A (2010) Optic nerve and vitreal inflammation are both RGC neuroprotective but only the latter is RGC axogenic. Neurobiol Dis 37:441-454. 10.1016/j.nbd.2009.10.024 19900554

[B6] Badylak S, Meurling S, Chen M, Spievack A, Simmons-Byrd A (2000) Resorbable bioscaffold for esophageal repair in a dog model. J Pediatr Surg 35:1097-1103. 10.1053/jpsu.2000.7834 10917304

[B7] Badylak SF (2004) Xenogeneic extracellular matrix as a scaffold for tissue reconstruction. Transpl Immunol 12:367-377. 10.1016/j.trim.2003.12.016 15157928

[B8] Badylak SF (2007) The extracellular matrix as a biologic scaffold material. Biomaterials 28:3587-3593. 10.1016/j.biomaterials.2007.04.043 17524477

[B9] Badylak SF, Valentin JE, Ravindra AK, McCabe GP, Stewart-Akers AM (2008) Macrophage phenotype as a determinant of biologic scaffold remodeling. Tissue Eng Part A 14:1835-1842. 10.1089/ten.tea.2007.0264 18950271

[B10] Badylak SF, Vorp DA, Spievack AR, Simmons-Byrd A, Hanke J, Freytes DO, Thapa A, Gilbert TW, Nieponice A (2005) Esophageal reconstruction with ECM and muscle tissue in a dog model. J Surg Res 128:87-97. 10.1016/j.jss.2005.03.002 15922361

[B11] Baehr M, Bunge RP (1989) Functional status influences the ability of Schwann cells to support adult rat retinal ganglion cell survival and axonal regrowth. Exp Neurol 106:27-40. 2792296 10.1016/0014-4886(89)90141-6

[B12] Baiguera S, Del Gaudio C, Lucatelli E, Kuevda E, Boieri M, Mazzanti B, Bianco A, Macchiarini P (2014) Electrospun gelatin scaffolds incorporating rat decellularized brain extracellular matrix for neural tissue engineering. Biomaterials 35:1205-1214. 10.1016/j.biomaterials.2013.10.060 24215734

[B13] Bartus K, James ND, Didangelos A, Bosch KD, Verhaagen J, Yáñez-Muñoz RJ, Rogers JH, Schneider BL, Muir EM, Bradbury EJ (2014) Large-scale chondroitin sulfate proteoglycan digestion with chondroitinase gene therapy leads to reduced pathology and modulates macrophage phenotype following spinal cord contusion injury. J Neurosci 34:4822-4836. 10.1523/JNEUROSCI.4369-13.2014 24695702 PMC3972714

[B14] Berkelaar M, Clarke DB, Wang YC, Bray GM, Aguayo AJ (1994) Axotomy results in delayed death and apoptosis of retinal ganglion cells in adult rats. J Neurosci 14:4368-4374. 8027784 10.1523/JNEUROSCI.14-07-04368.1994 PMC6577016

[B15] Berry M, Ahmed Z, Lorber B, Douglas M, Logan A (2008) Regeneration of axons in the visual system. Restor Neurol Neurosci 26:147-174. 18820408

[B16] Berry M, Carlile J, Hunter A (1996) Peripheral nerve explants grafted into the vitreous body of the eye promote the regeneration of retinal ganglion cell axons severed in the optic nerve. J Neurocytol 25:147-170. 8699196 10.1007/BF02284793

[B17] Berthiaume F, Moghe PV, Toner M, Yarmush ML (1996) Effect of extracellular matrix topology on cell structure, function, and physiological responsiveness: hepatocytes cultured in a sandwich configuration. FASEB J 10:1471-1484. 8940293 10.1096/fasebj.10.13.8940293

[B18] Bible E, Dell'Acqua F, Solanky B, Balducci A, Crapo PM, Badylak SF, Ahrens ET, Modo M (2012) Non-invasive imaging of transplanted human neural stem cells and ECM scaffold remodeling in the stroke-damaged rat brain by (19)F- and diffusion-MRI. Biomaterials 33:2858-2871. 10.1016/j.biomaterials.2011.12.033 22244696 PMC3268910

[B19] Bissell MJ, Barcellos-Hoff MH (1987) The influence of extracellular matrix on gene expression: is structure the message? J Cell Sci Suppl 8:327-343. 3332665 10.1242/jcs.1987.supplement_8.18

[B20] Bray GM, Vidal-Sanz M, Aguayo AJ (1987) Regeneration of axons from the central nervous system of adult rats. Prog Brain Res 71:373-379. 3588955 10.1016/s0079-6123(08)61838-5

[B21] Brennan EP, Reing J, Chew D, Myers-Irvin JM, Young EJ, Badylak SF (2006) Antibacterial activity within degradation products of biological scaffolds composed of extracellular matrix. Tissue Eng 12:2949-2955. 10.1089/ten.2006.12.2949 17518662 PMC3056877

[B22] Brennan EP, Tang XH, Stewart-Akers AM, Gudas LJ, Badylak SF (2008) Chemoattractant activity of degradation products of fetal and adult skin extracellular matrix for keratinocyte progenitor cells. J Tissue Eng Regen Med 2:491-498. 10.1002/term.123 18956412 PMC2706581

[B23] Brinkmann V, Zychlinsky A (2012) Neutrophil extracellular traps: is immunity the second function of chromatin? J Cell Biol 198:773-783. 10.1083/jcb.201203170 22945932 PMC3432757

[B24] Brown BN, Londono R, Tottey S, Zhang L, Kukla KA, Wolf MT, Daly KA, Reing JE, Badylak SF (2012) Macrophage phenotype as a predictor of constructive remodeling following the implantation of biologically derived surgical mesh materials. Acta Biomater 8:978-987. 10.1016/j.actbio.2011.11.031 22166681 PMC4325370

[B25] Brown BN, Valentin JE, Stewart-Akers AM, McCabe GP, Badylak SF (2009) Macrophage phenotype and remodeling outcomes in response to biologic scaffolds with and without a cellular component. Biomaterials 30:1482-1491. 10.1016/j.biomaterials.2008.11.040 19121538 PMC2805023

[B26] Butt AM, Pugh M, Hubbard P, James G (2004) Functions of optic nerve glia: axoglial signalling in physiology and pathology. Eye 18:1110-1121. 10.1038/sj.eye.6701595 15534596

[B27] Carey LE, Dearth CL, Johnson SA, Londono R, Medberry CJ, Daly KA, Badylak SF (2014) In vivo degradation of 14C-labeled porcine dermis biologic scaffold. Biomaterials 35:8297-8304. 10.1016/j.biomaterials.2014.06.015 24997479 PMC5372693

[B28] Charteris DG (1995) Proliferative vitreoretinopathy: pathobiology, surgical management, and adjunctive treatment. Br J Ophthalmol 79:953-960. 7488586 10.1136/bjo.79.10.953 PMC505299

[B29] Cherry JD, Olschowka JA, O'Banion MK (2014) Neuroinflammation and M2 microglia: the good, the bad, and the inflamed. J Neuroinflammation 11:98. 10.1186/1742-2094-11-98 24889886 PMC4060849

[B30] Cho MS, Rinker BD, Weber RV, Chao JD, Ingari JV, Brooks D, Buncke GM (2012) Functional outcome following nerve repair in the upper extremity using processed nerve allograft. J Hand Surg Am 37:2340-2349. 10.1016/j.jhsa.2012.08.028 23101532

[B31] Crapo PM, Medberry CJ, Reing JE, Tottey S, van der Merwe Y, Jones KE, Badylak SF (2012) Biologic scaffolds composed of central nervous system extracellular matrix. Biomaterials 33:3539-3547. 10.1016/j.biomaterials.2012.01.044 22341938 PMC3516286

[B32] Crapo PM, Tottey S, Slivka PF, Badylak SF (2014) Effects of biologic scaffolds on human stem cells and implications for CNS tissue engineering. Tissue Eng Part A 20:313-323. 10.1089/ten.TEA.2013.0186 24004192 PMC3875189

[B33] Crisan M, Yap S, Casteilla L, Chen CW, Corselli M, Park TS, Andriolo G, Sun B, Zheng B, Zhang L, Norotte C, Teng PN, Traas J, Schugar R, Deasy BM, Badylak S, Buhring HJ, Giacobino JP, Lazzari L, Huard J, et al (2008) A perivascular origin for mesenchymal stem cells in multiple human organs. Cell Stem Cell 3:301-313. 10.1016/j.stem.2008.07.003 18786417

[B34] Cruz-Guilloty F, Saeed AM, Echegaray JJ, Duffort S, Ballmick A, Tan Y, Betancourt M, Viteri E, Ramkhellawan GC, Ewald E, Feuer W, Huang D, Wen R, Hong L, Wang H, Laird JM, Sene A, Apte RS, Salomon RG, Hollyfield JG, Perez VL (2013) Infiltration of proinflammatory m1 macrophages into the outer retina precedes damage in a mouse model of age-related macular degeneration. Int J Inflam 2013:503725 10.1155/2013/503725 23533946 PMC3606733

[B35] Davis GE, Bayless KJ, Davis MJ, Meininger GA (2000) Regulation of tissue injury responses by the exposure of matricryptic sites within extracellular matrix molecules. Am J Pathol 156:1489-1498. 10.1016/S0002-9440(10)65020-1 10793060 PMC1876929

[B36] de Lima S, Koriyama Y, Kurimoto T, Oliveira JT, Yin Y, Li Y, Gilbert HY, Fagiolini M, Martinez AM, Benowitz L (2012) Full-length axon regeneration in the adult mouse optic nerve and partial recovery of simple visual behaviors. Proc Natl Acad Sci U S A 109:9149-9154. 10.1073/pnas.1119449109 22615390 PMC3384191

[B37] DeQuach JA, Yuan SH, Goldstein LS, Christman KL (2011) Decellularized porcine brain matrix for cell culture and tissue engineering scaffolds. Tissue Eng Part A 17:2583-2592. 10.1089/ten.tea.2010.0724 21883047 PMC3204197

[B38] Didangelos A, Iberl M, Vinsland E, Bartus K, Bradbury EJ (2014) Regulation of IL-10 by chondroitinase ABC promotes a distinct immune response following spinal cord injury. J Neurosci 34:16424-16432. 10.1523/JNEUROSCI.2927-14.2014 25471580 PMC4252552

[B39] Donnelly DJ, Popovich PG (2008) Inflammation and its role in neuroprotection, axonal regeneration and functional recovery after spinal cord injury. Exp Neurol 209:378-388. 10.1016/j.expneurol.2007.06.009 17662717 PMC2692462

[B40] Donnelly E, Lamanna J, Boulis N (2012) Stem cell therapy for the spinal cord. Stem Cell Res Ther 3:24. 10.1186/scrt115 22776143 PMC3580462

[B41] Edan RA, Luqmani YA, Masocha W (2013) COL-3, a chemically modified tetracycline, inhibits lipopolysaccharide-induced microglia activation and cytokine expression in the brain. PLoS One 8:e57827. 10.1371/journal.pone.0057827 23469077 PMC3585197

[B42] Eveleth DD (2013) Cell-based therapies for ocular disease. J Ocul Pharmacol Ther 29:844-854. 10.1089/jop.2013.0028 24050306

[B43] Fisher SK, Lewis GP, Linberg KA, Verardo MR (2005) Cellular remodeling in mammalian retina: results from studies of experimental retinal detachment. Prog Retin Eye Res 24:395-431. 10.1016/j.preteyeres.2004.10.004 15708835

[B44] Gao H, Zhang HL, Shou J, Chen L, Shen Y, Tang Q, Huang J, Zhu J (2012) Towards retinal ganglion cell regeneration. Regen Med 7:865-875. 10.2217/rme.12.97 23164085

[B45] Gao S, Zheng Y, Cai Q, Yao W, Wang J, Zhang P, Wang X (2014) Comparison of morphology and biocompatibility of acellular nerve scaffolds processed by different chemical methods. J Mater Sci Mater Med 25:1283-1291. 10.1007/s10856-014-5150-3 24452272

[B46] Gilbert TW, Sellaro TL, Badylak SF (2006) Decellularization of tissues and organs. Biomaterials 27:3675-3683. 10.1016/j.biomaterials.2006.02.014 16519932

[B47] Giulian D, Robertson C (1990) Inhibition of mononuclear phagocytes reduces ischemic injury in the spinal cord. Ann Neurol 27:33-42. 10.1002/ana.410270107 2301926

[B48] Godwin JW, Pinto AR, Rosenthal NA (2013) Macrophages are required for adult salamander limb regeneration. Proc Natl Acad Sci U S A 110:9415-9420. 10.1073/pnas.1300290110 23690624 PMC3677454

[B49] Godwin JW, Rosenthal N (2014) Scar-free wound healing and regeneration in amphibians: immunological influences on regenerative success. Differentiation 87:66-75. 10.1016/j.diff.2014.02.002 24565918

[B50] Goldberg JL, Klassen MP, Hua Y, Barres BA (2002) Amacrine-signaled loss of intrinsic axon growth ability by retinal ganglion cells. Science 296:1860-1864. 10.1126/science.1068428 12052959

[B51] Gris D, Marsh DR, Oatway MA, Chen Y, Hamilton EF, Dekaban GA, Weaver LC (2004) Transient blockade of the CD11d/CD18 integrin reduces secondary damage after spinal cord injury, improving sensory, autonomic, and motor function. J Neurosci 24:4043-4051. 10.1523/JNEUROSCI.5343-03.2004 15102919 PMC6729422

[B52] Guo SZ, Ren XJ, Wu B, Jiang T (2010) Preparation of the acellular scaffold of the spinal cord and the study of biocompatibility. Spinal Cord 48:576-581. 10.1038/sc.2009.170 20065987

[B53] Gupta N, Brown KE, Milam AH (2003) Activated microglia in human retinitis pigmentosa, late-onset retinal degeneration, and age-related macular degeneration. Exp Eye Res 76:463-471. 12634111 10.1016/s0014-4835(02)00332-9

[B54] Haan N, Zhu B, Wang J, Wei X, Song B (2015) Crosstalk between macrophages and astrocytes affects proliferation, reactive phenotype and inflammatory response, suggesting a role during reactive gliosis following spinal cord injury. J Neuroinflammmation 12:109. 10.1186/s12974-015-0327-3 26025034 PMC4457974

[B55] Hains BC, Waxman SG (2006) Activated microglia contribute to the maintenance of chronic pain after spinal cord injury. J Neurosci 26:4308-4317. 10.1523/JNEUROSCI.0003-06.2006 16624951 PMC6674010

[B56] Hao NB, Lü MH, Fan YH, Cao YL, Zhang ZR, Yang SM (2012) Macrophages in tumor microenvironments and the progression of tumors. Clin Dev Immunol 2012:948098. 10.1155/2012/948098 22778768 PMC3385963

[B57] Hauk TG, Müller A, Lee J, Schwendener R, Fischer D (2008) Neuroprotective and axon growth promoting effects of intraocular inflammation do not depend on oncomodulin or the presence of large numbers of activated macrophages. Exp Neurol 209:469-482. 10.1016/j.expneurol.2007.09.020 18021771

[B58] He L, Marneros AG (2013) Macrophages are essential for the early wound healing response and the formation of a fibrovascular scar. Am J Pathol 182:2407-2417. 10.1016/j.ajpath.2013.02.032 23602833 PMC3668032

[B59] Hertz J, Qu B, Hu Y, Patel RD, Valenzuela DA, Goldberg JL (2014) Survival and integration of developing and progenitor-derived retinal ganglion cells following transplantation. Cell Transplant 23:855-872. 10.3727/096368913X667024 23636049

[B60] Hodde JP, Record RD, Liang HA, Badylak SF (2001) Vascular endothelial growth factor in porcine-derived extracellular matrix. Endothelium 8:11-24. 11409848 10.3109/10623320109063154

[B61] Hong Y, Huber A, Takanari K, Amoroso NJ, Hashizume R, Badylak SF, Wagner WR (2011) Mechanical properties and in vivo behavior of a biodegradable synthetic polymer microfiber-extracellular matrix hydrogel biohybrid scaffold. Biomaterials 32:3387-3394. 10.1016/j.biomaterials.2011.01.025 21303718 PMC3184831

[B62] Horn KP, Busch SA, Hawthorne AL, van Rooijen N, Silver J (2008) Another barrier to regeneration in the CNS: activated macrophages induce extensive retraction of dystrophic axons through direct physical interactions. J Neurosci 28:9330-9341. 10.1523/JNEUROSCI.2488-08.2008 18799667 PMC2567141

[B63] Horne MK, Nisbet DR, Forsythe JS, Parish CL (2010) Three-dimensional nanofibrous scaffolds incorporating immobilized BDNF promote proliferation and differentiation of cortical neural stem cells. Stem Cells Dev 19:843-852. 10.1089/scd.2009.0158 19831634

[B64] Hu Y, Leaver SG, Plant GW, Hendriks WTJ, Niclou SP, Verhaagen J, Harvey AR, Cui Q (2005) Lentiviral-mediated transfer of CNTF to Schwann cells within reconstructed peripheral nerve grafts enhances adult retinal ganglion cell survival and axonal regeneration. Mol Ther 11:906-915. 10.1016/j.ymthe.2005.01.016 15922961

[B65] Huang KF, Hsu WC, Chiu WT, Wang JY (2012) Functional improvement and neurogenesis after collagen-GAG matrix implantation into surgical brain trauma. Biomaterials 33:2067-2075. 10.1016/j.biomaterials.2011.11.040 22169139

[B66] Hyatt AJT, Wang D, van Oterendorp C, Fawcett JW, Martin KR (2014) Mesenchymal stromal cells integrate and form longitudinally-aligned layers when delivered to injured spinal cord via a novel fibrin scaffold. Neurosci Lett 569:12-17. 10.1016/j.neulet.2014.03.023 24680849 PMC4015360

[B67] Jahnavi S, Kumary TV, Bhuvaneshwar GS, Natarajan TS, Verma RS (2015) Engineering of a polymer layered bio-hybrid heart valve scaffold. Mater Sci Eng C Mater Biol Appl 51:263-273. 10.1016/j.msec.2015.03.009 25842134

[B68] Jones LL, Margolis RU, Tuszynski MH (2003) The chondroitin sulfate proteoglycans neurocan, brevican, phosphacan, and versican are differentially regulated following spinal cord injury. Exp Neurol 182:399-411. 12895450 10.1016/s0014-4886(03)00087-6

[B69] Kador KE, Alsehli HS, Zindell AN, Lau LW, Andreopoulos FM, Watson BD, Goldberg JL (2014) Retinal ganglion cell polarization using immobilized guidance cues on a tissue-engineered scaffold. Acta Biomater 10:4939-4946. 10.1016/j.actbio.2014.08.032 25194930 PMC4254021

[B70] Karabekmez FE, Duymaz A, Moran SL (2009) Early clinical outcomes with the use of decellularized nerve allograft for repair of sensory defects within the hand. Hand (N Y) 4:245-249. 10.1007/s11552-009-9195-6 19412640 PMC2724628

[B71] Kawamura K, Komohara Y, Takaishi K, Katabuchi H, Takeya M (2009) Detection of M2 macrophages and colony-stimulating factor 1 expression in serous and mucinous ovarian epithelial tumors. Pathol Int 59:300-305. 10.1111/j.1440-1827.2009.02369.x 19432671

[B72] Kayama M, Nakazawa T, Thanos A, Morizane Y, Murakami Y, Theodoropoulou S, Abe T, Vavvas D, Miller JW (2011) Heat shock protein 70 (HSP70) is critical for the photoreceptor stress response after retinal detachment via modulating anti-apoptotic Akt kinase. Am J Pathol 178:1080-1091. 10.1016/j.ajpath.2010.11.072 21356360 PMC3069883

[B73] Keane TJ, Londono R, Turner NJ, Badylak SF (2012) Consequences of ineffective decellularization of biologic scaffolds on the host response. Biomaterials 33:1771-1781. 10.1016/j.biomaterials.2011.10.054 22137126

[B74] Kerschensteiner M, Stadelmann C, Dechant G, Wekerle H, Hohlfeld R (2003) Neurotrophic cross-talk between the nervous and immune systems: implications for neurological diseases. Ann Neurol 53:292-304. 10.1002/ana.10446 12601697

[B75] Khaing ZZ, Milman BD, Vanscoy JE, Seidlits SK, Grill RJ, Schmidt CE (2011) High molecular weight hyaluronic acid limits astrocyte activation and scar formation after spinal cord injury. J Neural Eng 8:046033. 10.1088/1741-2560/8/4/046033 21753237

[B76] Kigerl KA, Gensel JC, Ankeny DP, Alexander JK, Donnelly DJ, Popovich PG (2009) Identification of two distinct macrophage subsets with divergent effects causing either neurotoxicity or regeneration in the injured mouse spinal cord. J Neurosci 29:13435-13444. 10.1523/JNEUROSCI.3257-09.2009 19864556 PMC2788152

[B77] Klassen H, Warfvinge K, Schwartz PH, Kiilgaard JF, Shamie N, Jiang C, Samuel M, Scherfig E, Prather RS, Young MJ (2008) Isolation of progenitor cells from GFP-transgenic pigs and transplantation to the retina of allorecipients. Cloning Stem Cells 10:391-402. 10.1089/clo.2008.0010 18729769 PMC2976663

[B78] Koh TJ, DiPietro LA (2011) Inflammation and wound healing: the role of the macrophage. Expert Rev Mol Med 13:e23. 10.1017/S1462399411001943 21740602 PMC3596046

[B79] Kurimoto T, Yin Y, Omura K, Gilbert HY, Kim D, Cen LP, Moko L, Kugler S, Benowitz LI (2010) Long-distance axon regeneration in the mature optic nerve: contributions of oncomodulin, cAMP, and pten gene deletion. J Neurosci 30:15654-15663. 10.1523/JNEUROSCI.4340-10.2010 21084621 PMC3001271

[B80] Lathrop KL, Steketee MB (2013) Mitochondrial dynamics in retinal ganglion cell axon regeneration and growth cone guidance. J Ocul Biol 1:9. 24616897 PMC3946936

[B81] Lemley CA, Han DP (2007) Endophthalmitis: a review of current evaluation and management. Retina 27:662-680. 10.1097/IAE.0b013e3180323f96 17621174

[B82] Leon S, Yin Y, Nguyen J, Irwin N, Benowitz LI (2000) Lens injury stimulates axon regeneration in the mature rat optic nerve. J Neurosci 20:4615-4626. 10844031 10.1523/JNEUROSCI.20-12-04615.2000 PMC6772462

[B83] Lewis GP, Chapin EA, Luna G, Linberg KA, Fisher SK (2010) The fate of Müller's glia following experimental retinal detachment: nuclear migration, cell division, and subretinal glial scar formation. Mol Vis 16:1361-1372. 20664798 PMC2905639

[B84] Li C, Zhang X, Cao R, Yu B, Liang H, Zhou M, Li D, Wang Y, Liu E (2012) Allografts of the acellular sciatic nerve and brain-derived neurotrophic factor repair spinal cord injury in adult rats. PLoS One 7:e42813. 10.1371/journal.pone.0042813 22952613 PMC3429476

[B85] Lin CM, Lin JW, Chen YC, Shen HH, Wei L, Yeh YS, Chiang YH, Shih R, Chiu PL, Hung KS, Yang LY, Chiu WT (2009) Hyaluronic acid inhibits the glial scar formation after brain damage with tissue loss in rats. Surg Neurol 72 [Suppl 2]:S50–S54. 10.1016/j.wneu.2009.09.004 19944826

[B86] Liu J, Chen J, Liu B, Yang C, Xie D, Zheng X, Xu S, Chen T, Wang L, Zhang Z, Bai X, Jin D (2013) Acellular spinal cord scaffold seeded with mesenchymal stem cells promotes long-distance axon regeneration and functional recovery in spinal cord injured rats. J Neurol Sci 325:127-136. 10.1016/j.jns.2012.11.022 23317924

[B87] Liu S, Zhang H, Zhang X, Lu W, Huang X, Xie H, Zhou J, Wang W, Zhang Y, Liu Y, Deng Z, Jin Y (2011) Synergistic angiogenesis promoting effects of extracellular matrix scaffolds and adipose-derived stem cells during wound repair. Tissue Eng Part A 17:725-739. 10.1089/ten.TEA.2010.0331 20929282

[B88] Lorber B, Berry M, Logan A (2008) Different factors promote axonal regeneration of adult rat retinal ganglion cells after lens injury and intravitreal peripheral nerve grafting. J Neurosci Res 86:894-903. 10.1002/jnr.21545 18074384

[B89] Luna G, Lewis GP, Banna CD, Skalli O, Fisher SK (2010) Expression profiles of nestin and synemin in reactive astrocytes and Müller cells following retinal injury: a comparison with glial fibrillar acidic protein and vimentin. Mol Vis 16:2511-2523. 21139996 PMC2997333

[B90] Mansour-Robaey S, Clarke DB, Wang YC, Bray GM, Aguayo AJ (1994) Effects of ocular injury and administration of brain-derived neurotrophic factor on survival and regrowth of axotomized retinal ganglion cells. Proc Natl Acad Sci U S A 91:1632-1636. 8127857 10.1073/pnas.91.5.1632 PMC43217

[B91] Mantovani A, Sica A, Sozzani S, Allavena P, Vecchi A, Locati M (2004) The chemokine system in diverse forms of macrophage activation and polarization. Trends Immunol 25:677-686. 10.1016/j.it.2004.09.015 15530839

[B92] Martinez FO, Gordon S (2014) The M1 and M2 paradigm of macrophage activation: time for reassessment. F1000Prime Rep 6:13. 10.12703/P6-13 24669294 PMC3944738

[B93] McDevitt CA, Wildey GM, Cutrone RM (2003) Transforming growth factor-beta1 in a sterilized tissue derived from the pig small intestine submucosa. J Biomed Mater Res A 67:637-640. 10.1002/jbm.a.10144 14566807

[B94] McKeon RJ, Jurynec MJ, Buck CR (1999) The chondroitin sulfate proteoglycans neurocan and phosphacan are expressed by reactive astrocytes in the chronic CNS glial scar. J Neurosci 19:10778-10788. 10594061 10.1523/JNEUROSCI.19-24-10778.1999 PMC6784959

[B95] Mead B, Logan A, Berry M, Leadbeater W, Scheven BA (2013) Intravitreally transplanted dental pulp stem cells promote neuroprotection and axon regeneration of retinal ganglion cells after optic nerve injury. Invest Ophthalmol Vis Sci 54:7544-7556. 10.1167/iovs.13-13045 24150755

[B96] Mead B, Logan A, Berry M, Leadbeater W, Scheven BA (2014) Paracrine-mediated neuroprotection and neuritogenesis of axotomised retinal ganglion cells by human dental pulp stem cells: comparison with human bone marrow and adipose-derived mesenchymal stem cells. PLoS One 9:e109305. 10.1371/journal.pone.0109305 25290916 PMC4188599

[B97] Medberry CJ, Crapo PM, Siu BF, Carruthers CA, Wolf MT, Nagarkar SP, Agrawal V, Jones KE, Kelly J, Johnson SA, Velankar SS, Watkins SC, Modo M, Badylak SF (2013) Hydrogels derived from central nervous system extracellular matrix. Biomaterials 34:1033-1040. 10.1016/j.biomaterials.2012.10.062 23158935 PMC3512573

[B98] Medberry CJ, Tottey S, Jiang H, Johnson SA, Badylak SF (2012) Resistance to infection of five different materials in a rat body wall model. J Surg Res 173:38-44. 10.1016/j.jss.2010.08.035 20888581

[B99] Meng F, Modo M, Badylak SF (2014) Biologic scaffold for CNS repair. Regen Med 9:367-383. 10.2217/rme.14.9 24935046

[B100] Miyata T, Iizasa H, Sai Y, Fujii J, Terasaki T, Nakashima E (2005) Platelet-derived growth factor-BB (PDGF-BB) induces differentiation of bone marrow endothelial progenitor cell-derived cell line TR-BME2 into mural cells, and changes the phenotype. J Cell Physiol 204:948-955. 10.1002/jcp.20362 15828021

[B101] Mokarram N, Merchant A, Mukhatyar V, Patel G, Bellamkonda RV (2012) Effect of modulating macrophage phenotype on peripheral nerve repair. Biomaterials 33:8793-8801. 10.1016/j.biomaterials.2012.08.050 22979988 PMC3483037

[B102] Mosser DM, Edwards JP (2008) Exploring the full spectrum of macrophage activation. Nat Rev Immunol 8:958-969. 10.1038/nri2448 19029990 PMC2724991

[B103] Muller A, Hauk TG, Fischer D (2007) Astrocyte-derived CNTF switches mature RGCs to a regenerative state following inflammatory stimulation. Brain 130:3308-3320. 10.1093/brain/awm257 17971355

[B104] National Federation of the Blind (2013) Blindness statistics. Baltimore, MD: National Federation of the Blind.

[B105] Nelson CM, Bissell MJ (2006) Of extracellular matrix, scaffolds, and signaling: tissue architecture regulates development, homeostasis, and cancer. Annu Rev Cell Dev Biol 22:287-309. 10.1146/annurev.cellbio.22.010305.104315 16824016 PMC2933192

[B106] Ning B, Zhang A, Song H, Gong W, Ding Y, Guo S, Zhao Y, Jiang J, Jia T (2011) Recombinant human erythropoietin prevents motor neuron apoptosis in a rat model of cervical sub-acute spinal cord compression. Neurosci Lett 490:57-62. 10.1016/j.neulet.2010.12.025 21167907

[B107] Ozaki S, Radeke MJ, Anderson DH (2000) Rapid upregulation of fibroblast growth factor receptor 1 (flg) by rat photoreceptor cells after injury. Invest Ophthalmol Vis Sci 41:568-579. 10670490

[B108] Pascolini D, Mariotti SP (2012) Global estimates of visual impairment: 2010. Br J Ophthalmol 96:614-618. 10.1136/bjophthalmol-2011-300539 22133988

[B109] Penha EM, Meira CS, Guimarães ET, Mendonça MV, Gravely FA, Pinheiro CM, Pinheiro TM, Barrouin-Melo SM, Ribeiro-Dos-Santos R, Soares MB (2014) Use of autologous mesenchymal stem cells derived from bone marrow for the treatment of naturally injured spinal cord in dogs. Stem Cells Int 2014:437521. 10.1155/2014/437521 24723956 PMC3956412

[B110] Penn JS, McCollum GW, Barnett JM, Werdich XQ, Koepke KA, Rajaratnam VS (2006) Angiostatic effect of penetrating ocular injury: role of pigment epithelium-derived factor. Invest Ophthalmol Vis Sci 47:405-414. 10.1167/iovs.05-0673 16384991

[B159] Perniconi B, Costa A, Aulino P, Teodori L, Adamo S, Coletti D (2011) The pro-myogenic environment provided by whole organ scale acellular scaffolds from skeletal muscle. Biomaterials 32:7870-7882. 10.1016/j.biomaterials.2011.07.016 21802724

[B111] Popovich PG, Longbrake EE (2008) Can the immune system be harnessed to repair the CNS? Nat Rev Neurosci 9:481-493. 10.1038/nrn2398 18490917

[B112] Ramsden CM, Powner MB, Carr AJ, Smart MJ, da Cruz L, Coffey PJ (2013) Stem cells in retinal regeneration: past, present and future. Development 140:2576-2585. 10.1242/dev.092270 23715550 PMC3666384

[B113] Reing JE, Zhang L, Myers-Irvin J, Cordero KE, Freytes DO, Heber-Katz E, Bedelbaeva K, McIntosh D, Dewilde A, Braunhut SJ, Badylak SF (2009) Degradation products of extracellular matrix affect cell migration and proliferation. Tissue Eng Part A 15:605-614. 10.1089/ten.tea.2007.0425 18652541

[B114] National Center for Biotechnology Information (1997) Pieces of eight: bioactive fragments of extracellular proteins as regulators of angiogenesis. Trends Cell Biol 7:182-186. 10.1016/S0962-8924(97)01037-4 17708942

[B115] Schnell L, Fearn S, Klassen H, Schwab ME, Perry VH (1999) Acute inflammatory responses to mechanical lesions in the CNS: differences between brain and spinal cord. Eur J Neurosci 11:3648-3658. 10564372 10.1046/j.1460-9568.1999.00792.x

[B116] Schwab ME (2010) Functions of Nogo proteins and their receptors in the nervous system. Nat Rev Neurosci 11:799-811. 10.1038/nrn2936 21045861

[B117] Seiler MJ, Aramant RB (2012) Cell replacement and visual restoration by retinal sheet transplants. Prog Retin Eye Res 31:661-687. 10.1016/j.preteyeres.2012.06.003 22771454 PMC3472113

[B118] Sharp WW, Archer SL (2015) Mitochondrial dynamics in cardiovascular disease: fission and fusion foretell form and function. J Mol Med 93:225-228. 10.1007/s00109-015-1258-2 25669447 PMC4338002

[B119] Shechter R, London A, Varol C, Raposo C, Cusimano M, Yovel G, Rolls A, Mack M, Pluchino S, Martino G, Jung S, Schwartz M (2009) Infiltrating blood-derived macrophages are vital cells playing an anti-inflammatory role in recovery from spinal cord injury in mice. PLoS Med 6:e1000113. 10.1371/journal.pmed.1000113 19636355 PMC2707628

[B120] Shechter R, Miller O, Yovel G, Rosenzweig N, London A, Ruckh J, Kim KW, Klein E, Kalchenko V, Bendel P, Lira SA, Jung S, Schwartz M (2013) Recruitment of beneficial M2 macrophages to injured spinal cord is orchestrated by remote brain choroid plexus. Immunity 38:555-569. 10.1016/j.immuni.2013.02.012 23477737 PMC4115271

[B121] Sica A, Mantovani A (2012) Macrophage plasticity and polarization: in vivo veritas. J Clin Invest 122:787-795. 10.1172/JCI59643 22378047 PMC3287223

[B122] Sica A, Schioppa T, Mantovani A, Allavena P (2006) Tumour-associated macrophages are a distinct M2 polarised population promoting tumour progression: potential targets of anti-cancer therapy. Eur J Cancer 42:717-727. 10.1016/j.ejca.2006.01.003 16520032

[B123] Sicari BM, Johnson SA, Siu BF, Crapo PM, Daly KA, Jiang H, Medberry CJ, Tottey S, Turner NJ, Badylak SF (2012a) The effect of source animal age upon the in vivo remodeling characteristics of an extracellular matrix scaffold. Biomaterials 33:5524-5533. 10.1016/j.biomaterials.2012.04.017 22575834 PMC3569720

[B124] Sicari BM, Agrawal V, Siu BF, Medberry CJ, Dearth CL, Turner NJ, Badylak SF (2012b) A murine model of volumetric muscle loss and a regenerative medicine approach for tissue replacement. Tissue Eng Part A 18:1941-1948. 10.1089/ten.TEA.2012.0475 22906411 PMC3463275

[B125] Silver J, Miller JH (2004) Regeneration beyond the glial scar. Nat Rev Neurosci 5:146-156. 10.1038/nrn1326 14735117

[B126] Sipperley JO, Quigley HA, Gass DM (1978) Traumatic retinopathy in primates. The explanation of commotio retinae. Arch Ophthalmol 96:2267-2273. 718521 10.1001/archopht.1978.03910060563021

[B127] Soares HD, Hicks RR, Smith D, McIntosh TK (1995) Inflammatory leukocytic recruitment and diffuse neuronal degeneration are separate pathological processes resulting from traumatic brain injury. J Neurosci 15:8223-8233. 8613756 10.1523/JNEUROSCI.15-12-08223.1995 PMC6577921

[B128] Stankus JJ, Freytes DO, Badylak SF, Wagner WR (2008) Hybrid nanofibrous scaffolds from electrospinning of a synthetic biodegradable elastomer and urinary bladder matrix. J Biomater Sci Polym Ed 19:635-652. 10.1163/156856208784089599 18419942 PMC2860790

[B129] Sun F, Zhou K, Mi WJ, Qiu JH (2011) Combined use of decellularized allogeneic artery conduits with autologous transdifferentiated adipose-derived stem cells for facial nerve regeneration in rats. Biomaterials 32:8118-8128. 10.1016/j.biomaterials.2011.07.031 21816463

[B130] Tang S, Qiu J, Nikulina E, Filbin MT (2001) Soluble myelin-associated glycoprotein released from damaged white matter inhibits axonal regeneration. Mol Cell Neurosci 18:259-269. 10.1006/mcne.2001.1020 11591127

[B131] Thayer PS, Dimling AF, Plessl DS, Hahn MR, Guelcher SA, Dahlgren LA, Goldstein AS (2014) Cellularized cylindrical fiber/hydrogel composites for ligament tissue engineering. Biomacromolecules 15:75-83. 10.1021/bm4013056 24266805

[B132] Thiyagarajan M, Gonzales XF, Anderson H (2013) Regulated cellular exposure to non-thermal plasma allows preferentially directed apoptosis in acute monocytic leukemia cells. Stud Health Technol Inform 184:436-442. 23400198

[B133] Trimmer PA, Wunderlich RE (1990) Changes in astroglial scar formation in rat optic-nerve as a function of development. J Comp Neurol 296:359-378. 10.1002/cne.902960303 2358542

[B134] Turner JE, Blair JR, Chappell ET (1986) Peripheral nerve implantation into a penetrating lesion of the eye: stimulation of the damaged retina. Brain Res 376:246-254. 3730835 10.1016/0006-8993(86)90186-1

[B160] Turner NJ, Badylak JS, Weber DJ, Badylak SF (2012) Biologic scaffold remodeling in a dog model of complex musculoskeletal injury. J Surg Res 176:490-502. 10.1016/j.jss.2011.11.1029 22341350

[B135] Turner NJ, Badylak SF (2013) Biologic scaffolds for musculotendinous tissue repair. Eur Cell Mater 25:130-143. 23329468 10.22203/ecm.v025a09

[B136] Valentin JE, Stewart-Akers AM, Gilbert TW, Badylak SF (2009) Macrophage participation in the degradation and remodeling of extracellular matrix scaffolds. Tissue Eng Part A 15:1687-1694. 10.1089/ten.tea.2008.0419 19125644 PMC2792102

[B137] Valentin JE, Turner NJ, Gilbert TW, Badylak SF (2010) Functional skeletal muscle formation with a biologic scaffold. Biomaterials 31:7475-7484. 10.1016/j.biomaterials.2010.06.039 20638716 PMC2922042

[B138] Vasudevan S, Huang J, Botterman B, Matloub HS, Keefer E, Cheng J (2014) Detergent-free decellularized nerve grafts for long-gap peripheral nerve reconstruction. Plast Reconstr Surg Glob Open 2:e201. 10.1097/GOX.0000000000000118 25426384 PMC4236362

[B139] Vazquez-Chona F, Song BK, Geisert EE Jr (2004) Temporal changes in gene expression after injury in the rat retina. Invest Ophthalmol Vis Sci 45:2737-2746. 10.1167/iovs.03-1047 [Mismatch] 15277499 PMC2821791

[B140] Vidal-Sanz M, Bray GM, Villegas-Perez MP, Thanos S, Aguayo AJ (1987) Axonal regeneration and synapse formation in the superior colliculus by retinal ganglion cells in the adult rat. J Neurosci 7:2894-2909. 3625278 10.1523/JNEUROSCI.07-09-02894.1987 PMC6569122

[B141] Wang JY, Liou AK, Ren ZH, Zhang L, Brown BN, Cui XT, Badylak SF, Cai YN, Guan YQ, Leak RK, Chen J, Ji X, Chen L (2013) Neurorestorative effect of urinary bladder matrix-mediated neural stem cell transplantation following traumatic brain injury in rats. CNS Neurol Disord Drug Targets 12:413-425. 23469853 10.2174/1871527311312030014 PMC4049096

[B142] Wang L, Wang ZH, Shen CY, You ML, Xiao JF, Chen GQ (2010) Differentiation of human bone marrow mesenchymal stem cells grown in terpolyesters of 3-hydroxyalkanoates scaffolds into nerve cells. Biomaterials 31:1691-1698. 10.1016/j.biomaterials.2009.11.053 19962755

[B143] Wang T, Lai JH, Han LH, Tong X, Yang F (2014) Chondrogenic differentiation of adipose-derived stromal cells in combinatorial hydrogels containing cartilage matrix proteins with decoupled mechanical stiffness. Tissue Eng Part A 20:2131-2139. 10.1089/ten.tea.2013.0531 24707837

[B144] Weichel ED, Colyer MH (2008) Combat ocular trauma and systemic injury. Current Opin Ophthalmol 19:519-525. 10.1097/ICU.0b013e3283140e98 18854697

[B145] Wen R, Song Y, Cheng T, Matthes MT, Yasumura D, LaVail MM, Steinberg RH (1995) Injury-induced upregulation of bFGF and CNTF mRNAS in the rat retina. J Neurosci 15:7377-7385. 7472491 10.1523/JNEUROSCI.15-11-07377.1995 PMC6578062

[B146] Wittmer CR, Claudepierre T, Reber M, Wiedemann P, Garlick JA, Kaplan D, Egles C (2011) Multifunctionalized electrospun silk fibers promote axon regeneration in central nervous system. Adv Funct Mater 21:4202. 10.1002/adfm.201190103 22844266 PMC3404853

[B147] Wolf MT, Daly KA, Reing JE, Badylak SF (2012) Biologic scaffold composed of skeletal muscle extracellular matrix. Biomaterials 33:2916-2925. 10.1016/j.biomaterials.2011.12.055 22264525 PMC5942557

[B148] Xu Y, Patnaik S, Guo X, Li Z, Lo W, Butler R, Claude A, Liu Z, Zhang G, Liao J, Anderson PM, Guan J (2014) Cardiac differentiation of cardiosphere-derived cells in scaffolds mimicking morphology of the cardiac extracellular matrix. Acta Biomater 10:3449-3462. 10.1016/j.actbio.2014.04.018 24769114 PMC6029687

[B149] Xue H, Zhang XY, Liu JM, Song Y, Li YF, Chen D (2013) Development of a chemically extracted acellular muscle scaffold seeded with amniotic epithelial cells to promote spinal cord repair. J Biomed Mater Res A 101:145-156. 10.1002/jbm.a.34311 22829497

[B150] Yang L, Long Q, Liu J, Tang H, Li Y, Bao F, Qin D, Pei D, Liu X (2015) Mitochondrial fusion provides an “initial metabolic complementation” controlled by mtDNA. Cell Mol Life Sci 72:2585-2598. 25708700 10.1007/s00018-015-1863-9 PMC11113443

[B151] Yin Y, Cui Q, Li Y, Irwin N, Fischer D, Harvey AR, Benowitz LI (2003) Macrophage-derived factors stimulate optic nerve regeneration. J Neurosci 23:2284-2293. 12657687 10.1523/JNEUROSCI.23-06-02284.2003 PMC6742044

[B152] Yoon H, Kim G (2010) Micro/nanofibrous scaffolds electrospun from PCL and small intestinal submucosa. J Biomater Sci Polym Ed 21:553-562. 10.1163/156856209X429166 20338091

[B153] Yoshida K, Muraki Y, Ohki K, Harada T, Ohashi T, Matsuda H, Imaki J (1995) C-fos gene expression in rat retinal cells after focal retinal injury. Invest Ophthalmol Vis Sci 36:251-254. 7822154

[B154] Yrjänheikki J, Keinänen R, Pellikka M, Hökfelt T, Koistinaho J (1998) Tetracyclines inhibit microglial activation and are neuroprotective in global brain ischemia. Proc Natl Acad Sci U S A 95:15769-15774. 9861045 10.1073/pnas.95.26.15769 PMC28119

[B155] Yuan L, Neufeld AH (2001) Activated microglia in the human glaucomatous optic nerve head. J Neurosci Res 64:523-532. 11391707 10.1002/jnr.1104

[B156] Zeng HY, Green WR, Tso MO (2008) Microglial activation in human diabetic retinopathy. Arch Ophthalmol 126:227-232. 10.1001/archophthalmol.2007.65 18268214

[B157] Zhang L, Zhang F, Weng Z, Brown BN, Yan H, Ma XM, Vosler PS, Badylak SF, Dixon CE, Cui XT, Chen J (2013) Effect of an inductive hydrogel composed of urinary bladder matrix upon functional recovery following traumatic brain injury. Tissue Eng Part A 19:1909-1918. 10.1089/ten.tea.2012.0622 23596981 PMC3726021

[B158] Zhang XY, Xue H, Liu JM, Chen D (2012) Chemically extracted acellular muscle: a new potential scaffold for spinal cord injury repair. J Biomed Mater Res A 100:578-587. 10.1002/jbm.a.33237 22213649

